# Discrimination Training with Multimodal Stimuli Changes Activity in the Mushroom Body of the Hawkmoth *Manduca sexta*

**DOI:** 10.1371/journal.pone.0032133

**Published:** 2012-04-11

**Authors:** Anna Balkenius, Bill Hansson

**Affiliations:** 1 Division of Chemical Ecology, Department of Plant Protection Biology, Swedish Agricultural University, Alnarp, Sweden; 2 Max Planck Institute for Chemical Ecology, Jena, Germany; Center for Genomic Regulation, Spain

## Abstract

**Background:**

The mushroom bodies of the insect brain play an important role in olfactory processing, associative learning and memory. The mushroom bodies show odor-specific spatial patterns of activity and are also influenced by visual stimuli.

**Methodology/Principal Findings:**

Functional imaging was used to investigate changes in the *in vivo* responses of the mushroom body of the hawkmoth *Manduca sexta* during multimodal discrimination training. A visual and an odour stimulus were presented either together or individually. Initially, mushroom body activation patterns were identical to the odour stimulus and the multimodal stimulus. After training, however, the mushroom body response to the rewarded multimodal stimulus was significantly lower than the response to the unrewarded unimodal odour stimulus, indicating that the coding of the stimuli had changed as a result of training. The opposite pattern was seen when only the unimodal odour stimulus was rewarded. In this case, the mushroom body was more strongly activated by the multimodal stimuli after training. When no stimuli were rewarded, the mushroom body activity decreased for both the multimodal and unimodal odour stimuli. There was no measurable response to the unimodal visual stimulus in any of the experiments. These results can be explained using a connectionist model where the mushroom body is assumed to be excited by olfactory stimulus components, and suppressed by multimodal configurations.

**Conclusions:**

Discrimination training with multimodal stimuli consisting of visual and odour cues leads to stimulus specific changes in the *in vivo* responses of the mushroom body of the hawkmoth.

## Introduction

The mushroom body of insects was first described more than 150 years ago [1]. Some years later a more detailed account of the honeybee mushroom body architecture was provided [2], and today we know that the general design is similar in most insects [3], [4]. The mushroom bodies consist of two bilaterally symmetric neuropils in the insect brain that receive input regarding different sensory modalities.

Although most knowledge of the mushroom body relates to the olfactory system [5], it has previously been shown that the activity of the mushroom body can reflect an interaction between vision and olfaction [6]. The responses to bimodal stimuli consisting of odour and colour were recorded using calcium-sensitive optical imaging in the mushroom body of the hawkmoth *Manduca sexta*, showing that the activity in the mushroom body was influenced by both olfaction and vision. Colour could either enhance or suppress different odour-induced responses in the mushroom body of these hawkmoths. The multi-modal signal was also faster to influence the mushroom body than uni-modal stimuli [6]. These results can be compared to behavioural experiments that have shown that complex interactions occur between visual and olfactory stimuli. These interactions depend on the particular colours and odours used [7].

The mushroom body is also important for learning. Rapid and flexible learning to associate a colour or an odour individually with a reward has been demonstrated in honeybees, butterflies and moths [8]–[17]. In honeybees, three pairings of stimulus and reward are enough to retain the association for life [13], while a single trial can be enough for hawkmoths [18]. It has been shown in *Drosophila* that the mushroom body is necessary for olfactory associative learning [19]–[25]. During classical conditioning in honeybees, activation in the mushroom bodies was enhanced when one of the odours was rewarded [26]. The cellular response properties in the brain also changed during learning [27], [26]. However, the role of the mushroom body in visual processing is much less understood, although it has been shown that intact mushroom bodies are required for experience-dependent visual cognition in *Drosophila* [28].

There are several ways to train animals to distinguish different stimuli. In a discrimination experiment [29]–[31], the animals are presented with two different stimuli, one at a time, but only one of them is rewarded. As the result of repeated training with the two stimuli, the animal should learn to respond only to the stimulus that has been rewarded. During training, the animals will initially respond to both stimuli but as the learning progresses the response to the unrewarded stimuli vanishes. Hawkmoths can easily learn such discriminations [7], [32], [33].

Configural learning has been demonstrated in insects using classical as well as instrumental conditioning [34], [35]. When configural stimuli are used that consist of several stimulus components, for example a colour A and an odour B, several learning protocols become possible. The protocol where only the configural stimulus AB is rewarded, and A and B on their own are not (A/B/AB+), is called *positive patterning* [36]. Such a discrimination is often learned relatively quickly.

A discrimination that is more difficult to learn is called *negative patterning*, where only the individual components A and B are rewarded but not the multimodal stimulus AB. It is also possible to train animals to perform discriminations that are not symmetrical. The training protocol A/B+/AB is a form of *feature negative discrimination* that can be interpreted as meaning that the feature A signals that the target stimulus B is not rewarded [37].

We have in earlier experiments shown that free-flying hawkmoths can learn combinations of stimuli [7], which suggests that they are capable of configural learning [38]. We have also shown that *M. sexta* are fast learners and learn a previously unpreferred colour after as little as one single rewarded trial [18]. In the present study we were interested in investigating discrimination learning in real time in the mushroom body. We hypothesised that because the mushroom body is involved in odour processing, it should become increasingly active for odour stimuli that have been rewarded during training, while the activity should decrease for odour stimuli that were not rewarded. Our previous studies have shown that even though the mushroom body does not show any significant activation by visual stimuli, odour-evoked responses can still be modulated by these [6]. We expected this modulation to change depending on which stimulus was rewarded during discrimination training.

To address these questions, we used functional imaging *in vivo* to measure calcium dynamics in the calyx of the mushroom body during discrimination training, where visual, olfactory and rewarding sugar stimuli were presented to the animals in different combinations. The fully automated technique made detailed temporal control of the stimuli possible, while simultaneously recording how neural activation changed with each trial. The results suggest that the responses to multimodal stimuli in the mushroom body of the hawkmoth are tuned by learning and that a reward is necessary to maintain responses in the mushroom body. These activity changes can be explained by a connectionist model.

## Results

### Differential response decrease to a rewarded multimodal stimulus

**Figure 1:**
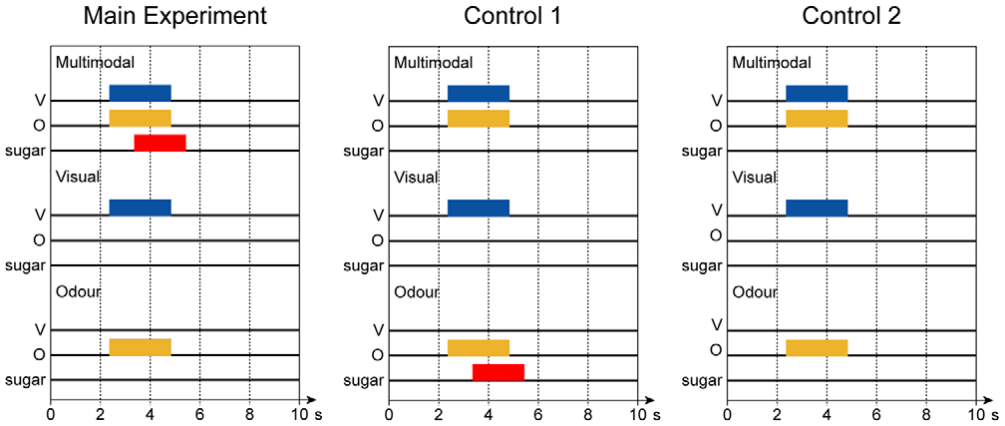
Stimulus timing. The timing of the three stimulus configurations and the sugar rewards for the three experiments. In the main experiment the odour and the visual stimulus were presented simultaneously followed by the sugar reward. This was followed by individual presentations of the visual and odour stimuli. In the first control experiment, the reward followed the unimodal presentation of the odour instead. Finally, in the second control experiment, the stimuli where presented without reward.

In the main experiment, moths were presented with three types of stimuli: unimodal presentation of the visual stimulus (V), unimodal presentation of an odour (O), and multimodal presentation of the visual stimulus together with odour (VO). The moths were rewarded during the presentation of the multimodal stimulus (Fig. 1A–C), but did not receive any reward with the unimodal stimuli, resulting in a positive patterning protocol (V/O/VO*+*).

**Figure 2:**
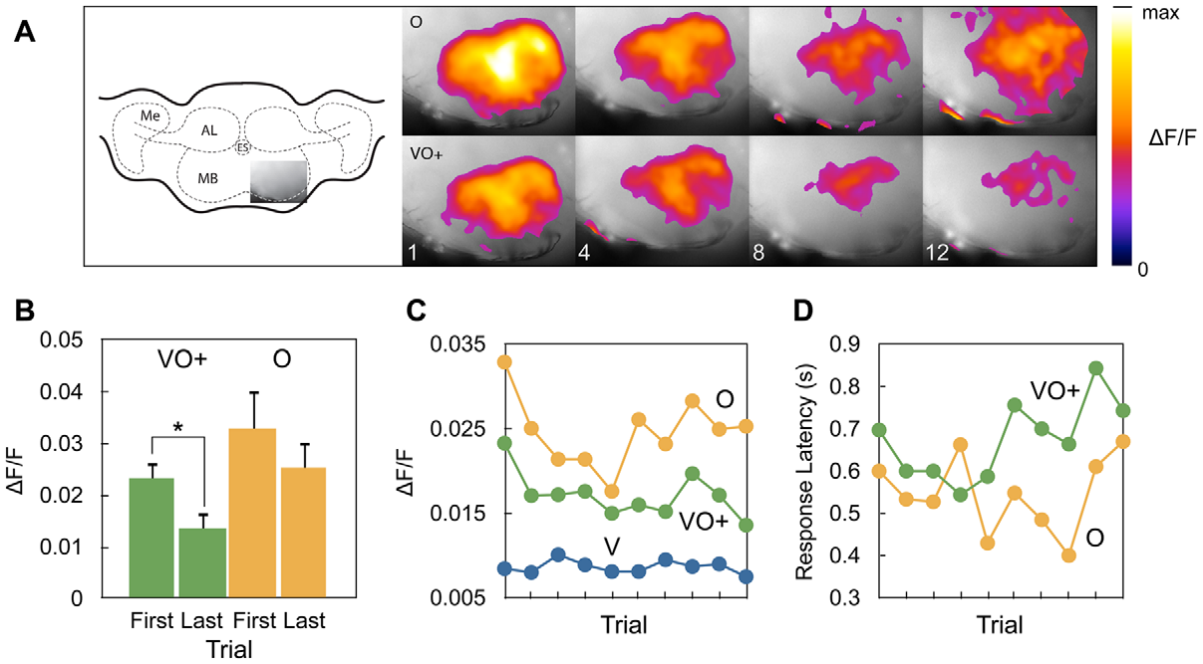
Activity changes during discrimination learning. A. The imaged location of the mushroom and the recorded signals at trial 1, 4, 8, and 12 for a single animal (Me: medulla, AL: antennal lobe, MB: mushroom body, Es: esophagus). B. The mean response of the mushroom body of all animals at the first and last training trials. The error bars show the standard error. C. The development of the activity of the mushroom body as a response to an unrewarded unimodal visual stimulus (V), an unrewarded unimodal odour stimulus (O), and a rewarded multimodal stimulus (VO+). Each point represents the median response of all animals in one trial. D. The latency of the mushroom body response to the multimodal and unimodal odour stimuli.

At the first trial, the measured mushroom body activation was not significantly different for the unrewarded odour stimulus (O) compared to the rewarded multimodal stimulus (VO+; Fig. 2A and B) (Mann-Whitney U=1640, n_1_=89, n_2_=41, p=0.36), which shows that there was no preference for the multimodal stimulus before training. However, after 10 trials, the response to VO+ became significantly lower than at the first trial (Mann-Whitney U=1509, n_1_=89, n_2_=27, p<0.05) indicating that learning changes the activity in the mushroom body (Fig. 2A and B). In contrast, there is no significant difference in the response to O (Mann-Whitney U=550, n_1_=41, n_2_=25, p=0.62).

After the first trial, the activity for both O and VO+ decreased, but after approximately 6 trials the curves began to diverge (Fig. 2C). The curve for VO+ activity showed a significant negative linear trend (F(1, 475)=7.963, p<0.01, b=−0.001), but this was not the case for the unrewarded stimulus O (F(1, 359)=0.03423, p=0.85).

The activity after presentation of the uni-modal unrewarded visual stimulus (V) remained low and showed no response (Fig. 2C). No significant differences in the motion of the images for the different conditions were observed excluding that the results were motion artefacts (Mann-Whitney U=38414, n_1_=227, n_2_=280, p=0.85). The median of all responses to each stimulus and the first and third quartile in the main experiment were VO+: [0.0093, 0.018, 0.033], O: [0.012, 0.024, 0.046], V: [0.0059, 0.0087, 0.011].

Also the onset of the response to the rewarded multimodal stimulus (VO+) and the uni-modal unrewarded odour stimulus (O) changed over the trials (Fig. 2D). The onset of the response to VO+ gradually became slower, while the onset to O got slightly faster with time (ANOVA,F(2, 969)=7.446, p<0.001).

At the end of the main experiment, 73% of the animals showed a proboscis extension reflex (PER) to the previously rewarded stimulus.

### Differential response decrease to a rewarded odour stimulus

The second group of moths were used as a control group. They were rewarded only during the presentation of the odour stimulus, and did not receive any reward with the multimodal or unimodal visual stimuli (V/O+/VO, Fig. 1A–C).

**Figure 3:**
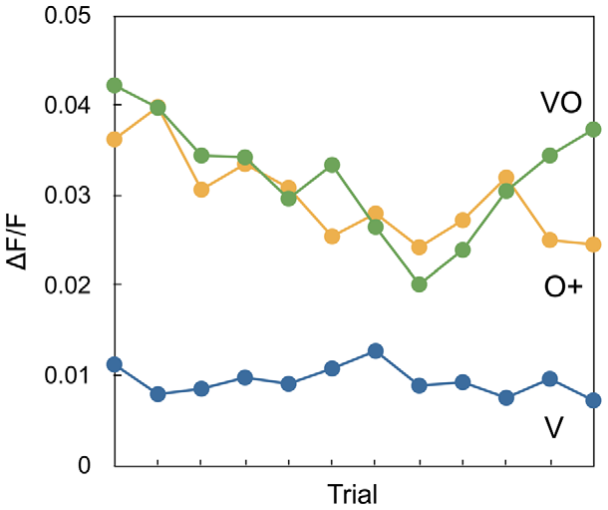
Activity changes with a rewarded odour. The development of the activity of the mushroom body as a response to an unrewarded unimodal visual stimulus (V), a rewarded unimodal odour stimulus (O+), and an unrewarded multimodal stimulus (VO). Each point represents a median response of all animals during a single trial.

The mushroom body activity elicited by the rewarded unimodal stimulus (O+), and by the bimodal stimulus (VO), started out at similar levels and gradually decreased in parallel during the first half of the experiment but then started to diverge (Fig. 3). There was no significant difference between the means for the two conditions (ANOVA, F(1, 553)=0.1663, p=0.68), but a significant interaction between the condition (O+, VO) and the activity change over time (ANOVA, F(2, 553)=5.7895, p<0.01) indicating that the training changed the mushroom body activity also in this experiment.

The activity for VO showed a weak but significant negative linear trend over all trials (F(1, 275)=4.182, p<0.05, b=−0.0010). However, analysing the slope of the activity change from trial 8 indicated a strong and significant positive linear trend for VO for the last five trials (F(1, 74) = 4.994, p<0.05, b=0.005). In contrast, the curve for O showed a significant negative linear trend over all trials (F(1, 275)=7.267, p<0.01, b=−0.0018).

As in the main experiment, the activity after presentation of the visual stimulus remained at the same level throughout the experiment (Fig. 3). There were no significant differences in the motion of the images for the different conditions excluding that the results were motion artefacts (Mann-Whitney U=38414, n_1_=277, n_2_=280, p=0.84). The median of all responses to each stimulus and the first and third quartile in the first control experiment were VO+: [0.017, 0.031, 0.046], O: [0.018, 0.030, 0.043], V: [0.0060, 0.0094, 0.017].

### Response decrease to unrewarded stimuli

In control experiment 2, we tested if the reward was necessary for the measured response changes in the mushroom body. The animals were presented with the same three stimuli as in the main experiment but no reward was given (V/O/VO).

**Figure 4:**
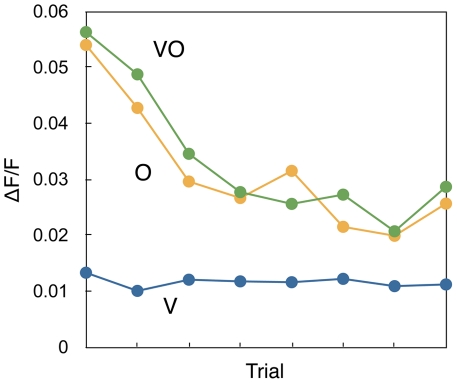
Activity changes without any reward. The development of the activity of the mushroom body as a response to an unrewarded unimodal visual stimulus (V), an unrewarded unimodal odour stimulus (O), and an unrewarded multimodal stimulus (VO). Each point represents a median response of all animals during a single trial.

The activity for the unimodal stimulus (O) and the bimodal stimulus (VO) started out at approximately the same level, and both decreased gradually over the trials (Fig. 4). There was no significant difference in the means for the two conditions (ANOVA, F(1, 271)=0.0032, p=0.95) and no interaction between the condition and the trials (F(2, 271)=2.9379, p=0.054). The curves for the two conditions both showed a significant negative linear trend (VO: F(1, 129)=10.65, p<0.01, b: −0.0033, O: F(1, 126)=12.63, p<0.001, b=−0.0032).

The activity of the visual stimulus remained at the same level during the experiment (Fig. 4). There were no significant differences in the motion of the images for the different conditions excluding that the results were motion artefacts (Mann-Whitney U=10604, n_1_=152, n_2_=147, p=0.45). The median of all responses to each stimulus and the first and third quartile in the second control experiment were VO+: [0.017, 0.030, 0.070], O: [0.019, 0.032, 0.074], V: [0.0091, 0.012, 0.020].

### Sugar Control

To make sure that the sugar reward did not produce the responses seen in the experiments, we tested five moths with only sugar and no colour or odour stimulus. The protocol was the same as for the main experiment but without any visual or odour stimulus. Measurements with only sugar reward were recorded and showed no response in the mushroom body.

## Discussion

Here we show that multimodal training with two modalities, odour and colour, changes the stimulus-induced activity in the mushroom body in the sphinx moth, *Manduca sexta*. For the mushroom body to exhibit a strong activation, it is necessary that the stimulus contains an odour component. A unimodal visual stimulus does not produce any measurable activity using our paradigm, although the visual component clearly influences the odour-induced activity in the mushroom body.

During training, the activity in the mushroom body for the stimulus configuration that is rewarded decreases over time (Fig. 2 and 3). The activity for the unrewarded stimulus also decreases initially, but starts to increase again at later trials. After training, the rewarded stimulus thus produces a weaker response than the unrewarded stimulus. The onset of the response for the rewarded stimulus configuration also increases over time (Fig. 2D). However, the stronger response for the unrewarded stimulus is only observed when at least one stimulus is rewarded. When no reward is involved at all, the activity decreases for all stimuli (Fig. 4).

**Figure 5:**
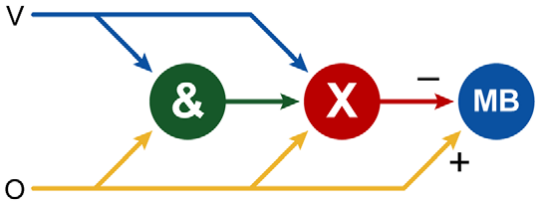
A connectionist model that explains the observed results. V: visual input, O: olfactory input, &: coding of the configural stimulus, X: the hypothesised region X, MB: mushroom body. The region X learns which stimulus configurations to associate with reward and subsequently suppresses the mushroom body. The mushroom body is directly activated by olfactory stimuli by the pathway from O to MB.

All the experimental results can be explained by a small number of assumptions that are summarised in the connectionist model in Figure 5. Connectionist models assume that measured responses can be explained by the operation of a number of units with connections of varying strengths that map the input signals to a response in a number of steps [39]. These connections are assumed to change their strengths during training.

First we assume that the mushroom body is activated initially by the olfactory stimulus through excitatory connections to the mushroom body. The presence of such connections, antennal lobe projection neurons, is well established [40]. A higher odour concentration leads to higher activation [41]. In the extension, this activation could be responsible for olfactory control of flight towards a nectar flower. If the presentation of the odour stimulus does not lead to a reward, these connections will be extinguished to make the animal less likely to approach that odour again [42]. This explains the result of control experiment 2, where the activity for both O and VO decreases over time (Fig. 4).

It is also clear that a unimodal visual stimulus will not produce any measurable response in the mushroom body (Fig. 2, 3, 4). Instead we assume that there is some other brain region X that controls visual responses, possibly part of the lateral protocerebrum [43]–[45]. We further assume that this region suppresses the odour response in the mushroom body, presumably to maintain visual control over the flight toward a flower [46]. Finally, we assume that activity in the region X reflects learning of the discrimination between unimodal and multimodal stimuli. To this end it receives a connection from a configural unit (&) that detects the presence of a multimodal stimulus. Although behavioural models of positive patterning usually do not need a configural unit, in this study the aim was to predict the activity of the mushroom body which makes a configural unit necessary to explain the result.

The result of the main experiment, where the multimodal stimulus was rewarded can now be explained: initially, both the unimodal odour stimulus and the multimodal stimulus activate the mushroom body since they have the odour component in common. The unimodal visual stimulus does not have any initial influence on the mushroom body.

During conditioning, the activity of region X gradually starts to discriminate between the rewarded multimodal stimulus and the two unrewarded unimodal stimuli. This learning can be explained by the type of model proposed by Kehoe [47], which can reproduce the temporal unfolding of discrimination learning (see also [30], [48]). Since the region X suppresses activation in the mushroom body, the measured activity will be lower for stimuli where region X is active, and as learning progresses, the activation of the mushroom body will decrease for the rewarded stimulus. The activity changes in the mushroom body we obtained in the current experiments for the pure odour and multimodal stimuli resembles classical learning curves for discrimination learning [46], except that they are inverted. This is consistent with inhibition of the mushroom body from a region that learns the discrimination. Since we have assumed that the mushroom body must be excited by an odour stimulus to show any activation, this also explains why there is no reaction to the unimodal visual stimulus.

Also the result of control experiment 1 is a direct consequence of this model. As in the main experiment, the mushroom body is initially activated by both the unimodal odour stimulus and the multimodal stimulus. As the odour is only rewarded when the visual stimulus is not present, the final activity after training is higher for the multimodal stimulus than for the unimodal odour stimulus, which is consistent with a stronger suppression from region X by the rewarded stimulus.

In control experiment 1, the suppression by the odour and multimodal stimuli is almost the same until the differential response becomes evident at the last two trials. This discrimination is thus learned more slowly compared to in the main experiment. This reflects the difference between the two types of learning in the main experiment and control 1. In the main experiment, the moths learn a positive patterning, which is the simplest form of multimodal interaction. In control experiment 1, however, they learn a (simultaneous) feature negative discrimination [36], which is typically much more difficult and involves both a configural unit and inhibitory learning [47].

Because the mushroom body is a site of integration of different modalities, and it has been shown in honeybee that sucrose or water elicited responses in the antennal lobe [49], we also tested if sucrose stimulation increased the calcium release. However, we did not observe any such responses in the mushroom body after sugar stimulation. Since the measured signal was recorded before the administration of the rewards in each trial, we could also rule out that the sugar reward was responsible for the different activity for different stimuli. In addition, the responses to our rewarded multimodal stimulus decreased over time excluding that the animals became aroused with the repeated rewards.

In conclusion, we have shown that discrimination training with multimodal stimuli consisting of visual and odour cues leads to changes in the *in vivo* responses of the mushroom body of the hawkmoth. The results can be explained by a connectionist model.

## Materials and Methods

### Animals

The animals used were both males and females of the hawkmoth *Manduca sexta* (Lepidoptera: Sphingidae). Larvae were reared on an artificial diet modified from Bell and Joachim [50] with 200 mg beta-carotene/l added [51]. The animals were kept under a 16 h∶8 h light/dark cycle at 23–25°C, 40–50% relative humidity. Experiments were performed on 2–4 days post-emergent naive moths.

The moths were secured in a plastic tube and fixed by dental wax. The head capsule was cut open between the eyes and neck. Muscle, glands and trachea were removed to expose the mushroom bodies. The eyes were covered by a flexible tube and fixed by dental wax. During recordings, light-guides were connected to the tubes. The proboscis was extended through a piece of flexible tubing, leaving the distal end of the proboscis exposed.

A calcium green-2-AM dye (Molecular Probes, Eugene) was dissolved in 20% Pluronic F-127 in dimethyl sulfoxide (Molecular Probes, Eugene) and diluted in moth Ringer solution to 30 µM. The calcium dye was applied directly to the brain and the preparation was left in a dark and cold (13°C) environment for 1–2 hours. Recordings were made *in vivo* after incubation and washing.

### Odour stimuli

The antennae were ventilated from a glass tube (7 mm internal diameter) with a continuous charcoal-filtered and moistened air stream (30 ml/s). The glass tube ended 10 mm from the antenna. The odourant consisting of phenylacetaldehyde (PAA) dissolved in paraffin oil was applied on filter paper (5×15 mm) and inserted into a Pasteur pipette [6]. The pipette was inserted trough a small hole in the continuous airflow glass tube with an air stream of 15 ml/s. Another air stream (5 m/s) was blown through the pipette by an automatically triggered puffer device (Syntech, Hilversum, The Netherlands) for 1 s into the continuous air stream. During odour stimulation, the air stream was switched from an empty pipette to an odour-laden one to minimise the influence of added air volume. Some animals were tested without any stimuli as a controls.

### Visual stimuli

The visual stimulus (V) was generated by a 3 mm LED of 430 nm and an intensity of approximately 0.01 cd/m^2^. This blue colour is known to be attractive to the moths during foraging [52]. The light source was controlled by a custom made interface, which controlled the intensity of the visual stimulus. A fiber-optic light guide was used to transfer the visual stimulus to the eyes of the moth. The optically isolated light guides where docked to the eyes using small rubber tubes that were kept in place using dental wax.

### Reward

The reward was a 20% sugar solution automatically administered by computer control. The animal was rewarded automatically using a container with the sugar solution that was moved toward the tip of the proboscis using a small servo-controlled micro-actuator to allow the animal to feed from the sugar solution.

### Optical recordings

An Olympus microscope was used for the measurement (filter settings: dichroic: 500 nm; emission LP 515 nm). The preparation was illuminated at 475 nm and responses were recorded through a 10x air objective (NA 0.50; Olympus, Hamburg, Germany). TILL Photonics imaging software (Gräfling, Germany) was used to record the brain responses and used sequences of 50 frames (4 Hz, 200 ms exposure time). The same regions of the calyces of the left and right mushroom bodies were recorded.

### Experimental procedure

Stimulus generation and data collection was fully automatic and controlled by the TILL-vision 4.0 software (TILL Photonics). Each trial consisted of three presentations using different protocols (Fig. 1): one with only vision (V), one with only odour (O) and one with both stimuli presented together (VO). These where always presented in the same order. The stimulus presentation started after 2.4 s and lasted for 2.4 s. On rewarded presentations, the sugar solution was offered 1 s later than the odour, colour or multimodal stimulus and lasted for 2 s. To avoid learning of temporal interval between the trails, the inter-trail interval was set to 180 s±120 s [53].

The number of animals in the main experiment was 47. The first control experiment used 27 animals and the second control experiment used 15 animals.

In control 3, five moths were not exposed to any visual or olfactory stimuli, but they were fed sugar solution to investigate if sugar would activate the mushroom body on its own. The experimental procedure for this sugar test group was identical to the other experiments except that no stimuli were presented.

### Data evaluation

The recorded images where first spatially filtered using a Gaussian filter to remove noise (σ=4.7 pixels). This was followed by an estimation of ΔF/F for each frame, where F was estimated using a linear function fitted to the parts of the Calcium fluorescence decay curve outside the potential response. The response magnitude was calculated as the average ΔF/F between the onset of the stimuli and the onset of the reward to avoid any influence of the sugar reward on the recorded signal. The latency of the response was set to the time of the first frame with a positive average ΔF/F after the stimulus presentation. The resolution of the onset measurement was thus identical to the frame rate. To check that the PER reflex did not produce motion in the recorded images would be incorrectly detected as calcium activity all image sequences where also analysed for motion using a block matching algorithms between successive frames.

For the statistical analysis, trial 1–10 were used for the main experiment, trial 1–12 were used for control experiment 1 and trial 1–8 were used for control experiment 2. The experiments continued as long as reliable signals could be recorded from the animals.

Mann-Whitney U Test was used to compare the responses to the different stimuli in the study since it is robust to outliers in the data. ANOVA was used to compare the slopes of the signal onset and magnitude change.
